# Dual-Scale Textured Broadband Si-Based Light Absorber

**DOI:** 10.3390/nano12234285

**Published:** 2022-12-01

**Authors:** Zhidong Wen, Shunshuo Cai, Zhe Zhang, Ziye Xu, Qi Song, Kunpeng Zhang, Man Li, Haiyan Shi, Yu Hou, Zichen Zhang

**Affiliations:** 1 Microelectronics Instruments and Equipment R&D Center, Institute of Microelectronics, Chinese Academy of Sciences, Beijing 100029, China; 2 School of Integrated Circuits, University of Chinese Academy of Sciences, Beijing 100049, China; 3 International Research Center for Nano Handling and Manufacturing of China, Changchun University of Science and Technology, Changchun 130022, China

**Keywords:** shape, dual-scale, FDTD, nanoripple-cone, femtosecond laser

## Abstract

Various antireflective structures and methods are proposed to solve the optical loss of Si-based absorber devices. Dual-scale structures have received more concern from researchers in recent years. In this study, the finite difference time domain (FDTD) method is employed to investigate deeply the dependence of optical response on the geometric shape and size of structures. The micron cone shows lower reflectivity than other micron structures. Additionally, the lowest reflectivity region moves with the increasing height size of the cone structure. We proposed creatively a nanoripple-cone structure that maintains low reflectivity properties under varying incident angles whether in the visible region or the near-infrared region. Furthermore, the lower reflectivity is obtained with increasing micron cone and decreasing nanoripple. Finally, the dual-scale nanoripple-cone is fabricated directly and cost-effectively by a femtosecond laser instead of a two-step texture-on-texture way. The measured result shows that the high absorption above 98% extends to the mid-infrared region. This study provides directions for the fabrication of wideband Si-based absorber devices to reduce reflectivity, which exhibits a wide application potential and promotes the evolution of multi-laser processing.

## 1. Introduction

The optical loss of silicon material is a critical factor to limit its application in Si-based optoelectronic devices due to its bandwidth. Various antireflective (AR) structures are designed to coat on the silicon surface, which is expected to reduce reflectance and enhance the efficiency of devices. The treated silicon sample is called “black silicon”, as the surface appears black to human eyes [1]. It has shown a wide application potential in reported studies, such as photo catalysis [2,3], photodetection [4,5], solar cell [6,7], Surface-Enhanced Raman Scattering (SERS) [8,9], sensing [10], et al.

A number of previous works have focused on the fabrication or modeling of AR structures and analyzed the corresponding optical responses. Several shapes of textures are proposed and prepared, including cone [11,12], nanowire [13,14], porous [15], pyramids [16], and so on. Nguyen et al. [17] prepared a nanoscale cone-shaped structure in a cryogenic deep reactive ion etching (DRIE) process with an O_2_–SF_6_ plasma. The reflectance of the treated sample was about 1% in the visible light regime. Mei et al. [18] produced cone-shaped microstructures by femtosecond laser and the reflectance was about 5% in the visible light region. In 2018, Zhang et al. [19] fabricated a honeycomb texture on the silicon surface. The reported average reflectance was about 20%, while the efficiency of the resultant solar cell was 18.57%. However, there is no deep study concerning the effect of different geometry shapes on the optical response of materials. Furthermore, the size of the structures from the nanoscale to the micron also affects the optical properties of the processed sample. In 2018, Sevin et al. [20] reported that a nanostructure with an aspect ratio of 4 was fabricated with cryogenic DRIE. Then, the b-Si solar cell achieved a conversion efficiency of 22.1%. Nguyen et al. [21] reported that the black silicon structure with sharp and high-density cones was expected to obtain the lowest reflectivity.

Nanoscale textures show drawbacks to subsequent processing, such as reduced absorption [22], interfacial interference [23], weak self-cleaning ability [24], and poor collection efficiency of photogenerated carriers due to increased surface recombination [25], though a high-aspect-ratio nanostructure is easily available. However, micron-scale structures have a lower aspect ratio despite that it is suitable for complex 3D devices [26]. The dual-scale structures have received attention in recent years due to their exhibits of a trade-off between optical response and subsequent processing [27]. Various hybrid structures were studied in a texture-on-texture way, such as nano porous–micron pyramid [7,28,29], nano cones–micron pyramid [30], nanopores–micropores [27], nanowires–micro pyramid [31], and nanopore–chimney [32]. In 2015, Andrea Ingenito et al. [30] fabricated the nano cone–micro pyramid by reactive ion etching–alkaline etching. The treated sample shows excellent absorption in the waveband of 300 nm–1000 nm while the conversion efficiency of solar cells is 19.8%. In 2022, Jiahui Xu et al. [33] prepared nanopores on top of a pre-formed alkaline-etched micron-pyramid surface (NPP) by RIE. The optical response of NPP structures outperforms the micro-pyramid alone. The average conversion efficiency of 23.55% and short circuit current density of 41.44 mA/cm^2^ have been achieved in the resultant solar cells. However, the texture-on-texture way is subjected to cost and processing time. Vorobyev et al. [34] produced nanostructure-textured microgrooves on silicon directly by femtosecond laser scanning. The average depth of microgrooves is 55 µm and the antireflection effect extends to the mid-infrared range. In 2020, Tong Chen et al. [35] fabricated a multi-scale micro-nano composite structure in ambient air by femtosecond laser. The reflectance below 5% from 2.5–10 µm is achieved.

In this work, we provide a deep insight for the dependence of optical response for black silicon on various geometric shapes and height sizes. The finite difference time domain (FDTD) method is introduced to simulate light radiation without the consideration of doping. Then, a dual-scale nanoripple-micron cone structure is proposed creatively, and we study the effects of micron cone height and nanoripple height, as well as the optical response of the nanoripple-cone under varying incident angles. The simulation results indicate that a better optical response is obtained with increasing the micron cone height and decreasing the nanoripple height. Furthermore, we fabricate the dual-scale structure directly by laser-inducing assisted with laser plasma shockwave cleaning in ambient air, which is an economical method compared to the texture-on-texture ways. The measured results show that the high absorption effect above 98% of the nanoripple-micron cone extends to mid-infrared. This work provides valuable directions for researchers and engineers about Si-based absorber devices.

## 2. Simulation and Fabrication

### 2.1. Simulation Setup

Generally, radiative energy absorption is expressed by Maxwell’s equations, which are used to calculate the interaction between the photons and the cell. The particular form of Maxwell’s Equations (1)–(3) is shown [36]:

(1) $$\frac{\partial \vec{D}}{\partial t}=\nabla \times \vec{H}$$

(2) $$\vec{D}\left(w\right)=\varepsilon_{0}\varepsilon_{r}\left(w\right)\vec{E}\left(w\right)$$

(3) $$\frac{\partial \vec{H}}{\partial t}=-\frac{1}{\mu_{0}}\nabla \times \vec{E}$$

where *H*, *E*, and *D* are the magnetic, electric, and displacement fields, respectively, while $\varepsilon_{r}\left(w\right)$ is the complex relative dielectric constant ($\varepsilon_{r}\left(w\right)=n^{2}$, where n is the refractive index).

A few methods have been employed to solve Maxwell’s equations, including the finite element method (FEM) [17], finite difference time domain (FDTD) [37], transfer matrix method (TMM) [38], and rigorous-coupled wave analysis (RCWA) [39]. In the previous literature [40], the FDTD method has shown high accuracy and robustness in the simulation of optical response over a wideband spectrum. Therefore, the commercial Lumerical FDTD solution software is used to study the influence of geometric shape and size of the coating structure on the optical properties of black silicon in this work. Furthermore, the optical response under varying incident angles is also investigated. 

**Figure 1:**
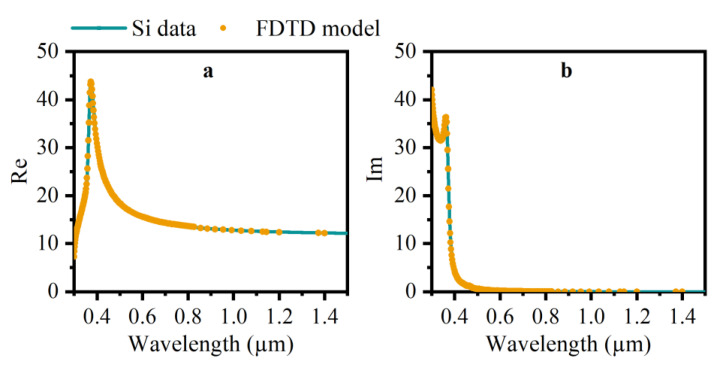
The fitted finite difference time domain (FDTD) model of refractive index and the material data of silicon ((**a**) silicon-n, (**b**) silicon-k).

To simplify simulation and reduce computational time, all structures are modeled in unit cells, though black silicon may not be a periodic structure array in reality. Doping in black silicon fabrication is not considered here. All unit cells of models in this simulation are outlined in the next section. The periodic boundary conditions are set in the x-y direction, while the broadband plane wave injects perpendicularly (incident angle 0°). However, to investigate the effect of varying incident angles with a fixed wavelength, the Bloch boundary conditions work in the x-y direction. Additionally, Perfectly Matched Layer conditions (PML) are set to keep accuracy in the z direction, respectively. A fitted model of refractive index (n, k), as shown in Figure 1, and other material data of silicon, which are referred to in the research of Palik [41], are working in this process. Additionally, the minimum length of the mesh cell is set to 0.25 nm. The termination criterion is 1 × 10^−5^ to confirm that the spectral response obtained by the Fourier transformation is valid. Three data monitors are placed to detect the optical response (Absorption-A, Reflectivity-R, Transmittance-T) of different simulated models.

### 2.2. Fabrication by Femtosecond Laser

**Figure 2:**
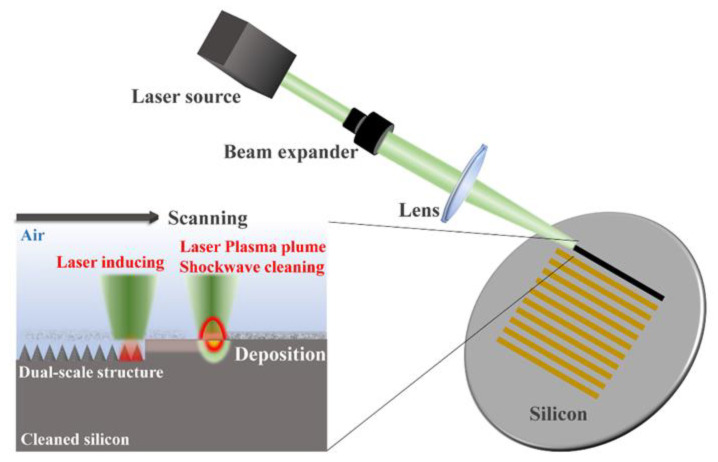
The schematic of experimental set up. The inset is a schematic diagram for the fabrication of black silicon.

Previously, the micro-nanoscale structures are usually prepared in a two-step texture-on-texture approach, such as alkaline etching+ reactive ion etching (RIE) [29,42] and RIE+ plasma etching [27]. In our work, we fabricate dual-scale nanoripple-cone structures directly on the silicon surface by a femtosecond laser without the addition of chemical contamination. Cleaned N-type silicon wafers (100) were used as samples commercially. A 25 W laser source (NKT Photonics, Copenhagen, Denmark) was employed to deliver 515 nm, 400 fs laser pulses with a repetition rate of 200 kHz. The laser spot with a diameter of 40 µm was irradiated on the silicon surface while the laser beam passes through the lens (focal length of 75 mm). The position of silicon samples was controlled precisely in a x-y-z platform with supporting software. The schematic of the experimental setup is illustrated in Figure 2. The schematic of the processing method, which is laser-inducing assisted with laser plasma shockwave cleaning in ambient air, is also presented.

The detailed mechanism to combine the laser-inducing and laser plasma shockwave cleaning technology has been presented in the previous work [43]. Herein, several parallel straight trajectories with a pitch of 18 µm were preliminarily designed. Firstly, each line was scanned three times back and forth using the laser cleaning technology to remove the deposition resulted from the previous scanned lines, while the substrate silicon was not damaged. Secondly, the cleaned modified line was processed using the laser-inducing technology to fabricate micro-nano structures. Finally, black silicon with a large area coated with nanoripple-micron cone structures was produced until the whole trajectories were finished. In the laser cleaning processing, the laser beam with a fluence of 0.3 J/cm^2^ was scanned at 20 mm/s, while in the inducing processing, the 1.8 J/cm^2^ laser beam scanned lines at 20 mm/s. Furthermore, to analyze the fabricated structures, the morphology of the treated sample was characterized by scanning electron microscope (SEM) (HITACHI, Tokyo, Japan). The spectral response measurements of dual-scale black silicon were undertaken using an ultraviolet spectrophotometer (for the waveband of 0.3–2.5 µm) (SHIMADZU, Kyoto, Japan) and a Bruker Tensor-Fourier transform infrared (FTIR) spectroscope equipped with integrating spheres (for the waveband of 2.5–15 µm).

## 3. Results and Discussion

### 3.1. Modeling Results of Various Micro/Nano Structures

#### 3.1.1. The Influence of Geometric Shapes

It is noted that the multireflection between microstructures plays an important role in reducing optical losses. The radiative transfer is generally expressed by Maxwell’s equation. Therefore, different geometric structures bring different optical responses. Various geometries have been modeled to investigate and analyze their corresponding results in this work, providing useful guidance for black silicon fabrication. In the simulation progress, it is necessary to simplify the shapes of modeled structures in unit cells due to the random fabricated structures and limited computational resources.

**Figure 3:**
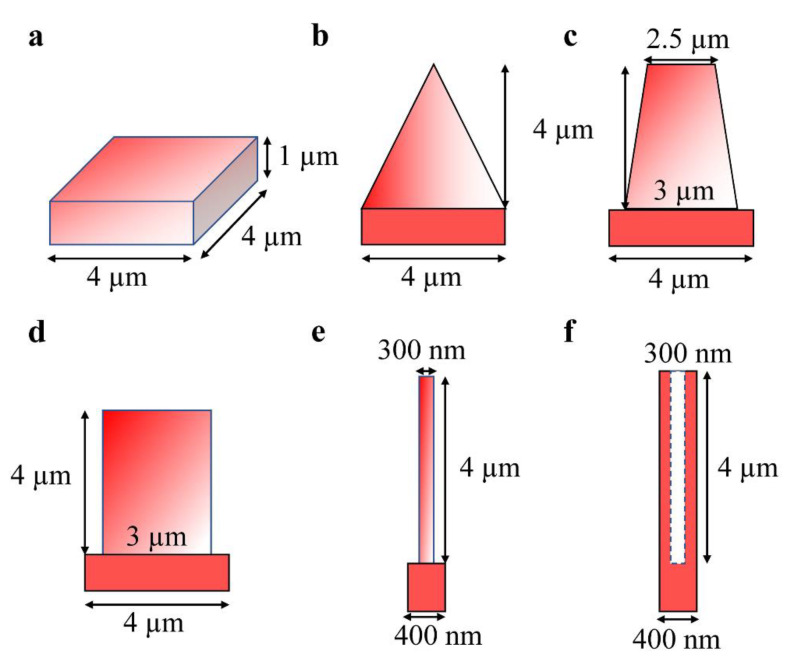
The detailed modeling information of unit cells about different microstructures, including: (**a**) planar silicon, (**b**) cone, (**c**) round column, (**d**) square column, (**e**) Si-NWAs and (**f**) Si-NHAs.

**Figure 4:**
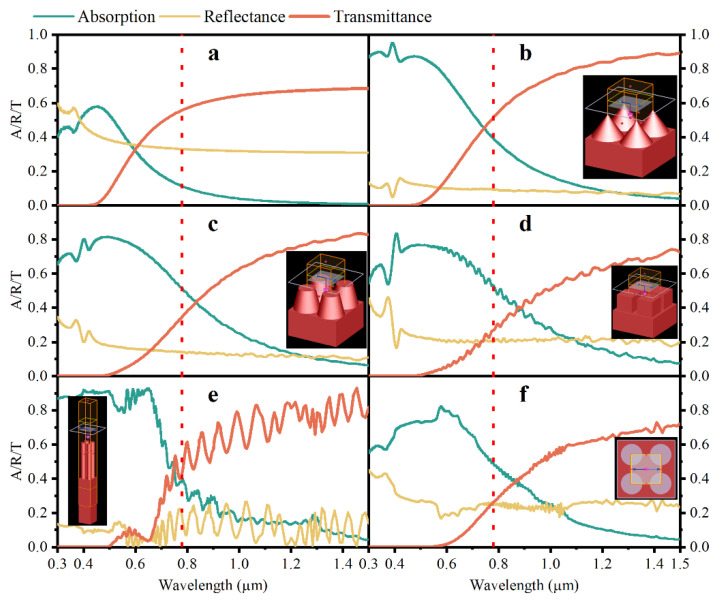
Optical response curves (absorption-A, reflectance-R, transmittance-T) of different microstructures shown in Figure 3, including (**a**) planar silicon, (**b**) cone, (**c**) round column, (**d**) square column, (**e**) Si-NWAs and (**f**) Si-NHAs. The inset corresponds to 3D models in simulation.

The microstructures reported in the literature [11,12,13,14,15,16] are investigated systematically. Figure 3 shows the detailed modeling information of unit cells about different micron structures (including planar silicon, cone, round column, square column, nanowires arrays (Si-NWAs) and nanohole (Si-NHAs)). Their optical response spectrums under the incident angle of 0°, including absorption (A), reflectance (R) and transmittance (T), are demonstrated, respectively, in Figure 4. The inset is the corresponding 3D models. The fixed height of all microstructures is 4 µm, while the fixed thickness of substrate is 1 µm. The periodicity of cone or round column or square column is 4 µm, while the Si-NWAs or Si-NHAs is 400 nm. Furthermore, the dimensions of diameters for all kinds of structures are equivalent to periodicity. 

Figure 4 proves that micron structures have a significant effect on enhancing optical absorption and reducing reflection. In Figure 4a, the absorption curve of planar silicon deteriorates from 0.6 to 0 as the wavelength increases from the visible light region to near infrared (NIR) and the reflectance decays to 0.3. The simulation results of planar silicon are close to the measured results reported by Zhang [27], which is helpful in confirming the validity of the simulation. Among various structures, cone arrays and Si-NWAs have exhibited the lowest reflectivity, which is about 0.1 in the waveband of 0.3–1.5 µm, while the reflectance curve in Figure 4b is flatter comparatively. In Figure 4b, the absorption is about 0.9 and decays to 0.36 as the wavelength is 0.8 µm due to the increasing transmittance. Therefore, micro cone arrays outperform (provide excellent optical performance) other geometry structures.

#### 3.1.2. The Influence of Height Size

**Figure 5:**
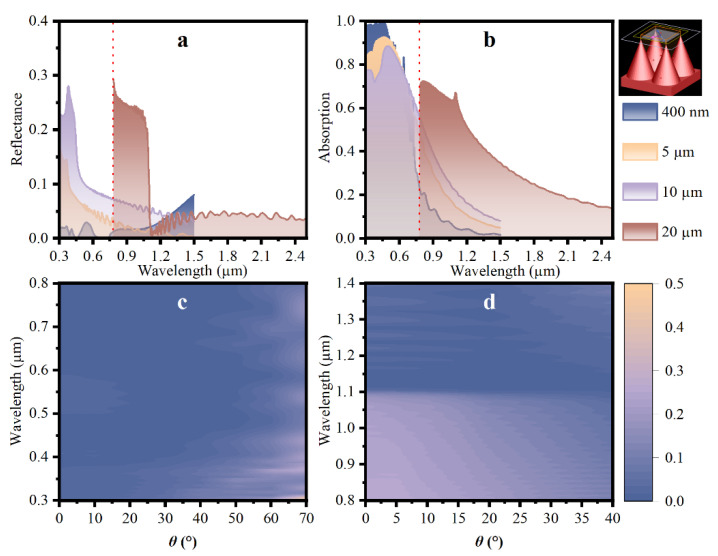
(**a**) Reflectance spectra and (**b**) absorptance spectra for structures with different heights (400 nm, 5 µm, 10 µm, 20 µm); reflectivity for structures with heights of (**c**) 400 nm and (**d**) 20 µm under varying incident angles as a function of wavelength.

The fabrication of structures with different sizes that yield different optical properties of black silicon depends on different processing methods or processing parameters. To further investigate the dependence of the reflection spectrum on the height size of the fabricated structure, different sizes of cone arrays (400 nm, 5 µm, 10 µm, 20 µm of height) were studied. All models have a fixed aspect ratio, meaning height:diameter of 2:1. The periodicity of the unit cell is equivalent to the diameter of the cone. The schematic of the 3D model is shown in the inset of Figure 5b.

The reflectance spectra and absorption spectra for cone arrays with different heights under the incident angle of 0° are illustrated, respectively, in Figure 5a,b. Figure 5a shows that the nanoscale cone exhibits the lowest reflectivity in the visible light region, and the curve tends to increase with the wavelength of the incident light, especially in the near-infrared region. For micron-scale structures, there is a decreasing trend to the lowest reflection waveband with an increasing wavelength. It is concluded that the lowest reflectance region of cone arrays moves along the light spectrum from visible light to near infrared (NIR), while the cone height increases from nanoscale to micron-scale. In Figure 5b, the absorption curve for structures of a 400 nm height falls faster than other curves for micron-scale structures, though it is close to 1 in a waveband of 0.3–0.45 µm. The average absorptance in the visible light spectrum is enhanced with the increasing height size of the structure. For the near-infrared region, the absorption curve for cone arrays with a height of 20 µm exhibits excellent optical properties due to the lowest reflectance performance. At the wavelength of 0.8 µm, the absorption for the 20 µm cone is 70% while the 400 nm scale falls to 20%.

Moreover, in order to analyze the influence of the incident angle on the reflectance of different scales of the structure, the optical response at different angles of incidence is studied for structures with a height of 400 nm and 20 µm. The reflectivity spectra for structures under varying incident angles as a function of wavelength are shown in Figure 5c,d. It is observed that the nanoscale cone shows low reflectance performance under varying incident angles mainly in the visible light regime, while the micron cone is in the NIR waveband. The low reflectivity for the cone of a height of 400 nm remains under the incident angle of 0-50°, as well as the 20 µm cone is in 0–40°.

#### 3.1.3. Dual-Textured Black Silicon

**Figure 6:**
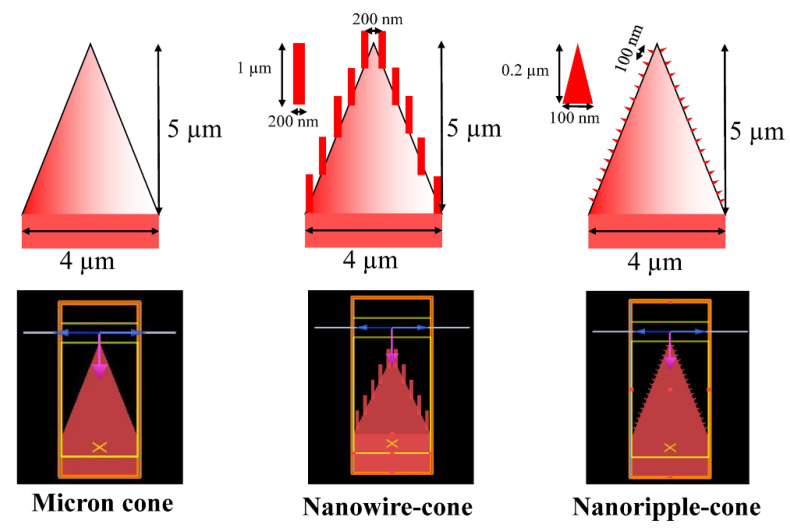
Modeling information and 2D models of cone alone, nanowire-cone and nanoripple-cone.

The micro-nano structure has attracted the interest of researchers to be applied to reduce broadband optical reflection due to the combination of graded reflective index of nanoscale and the multireflection of micro-scale. We have found that the lowest reflection region is different as the size of the structure increases according to the above simulation results. A nanoripple-micron cone structure fabricated on silicon is proposed to enhance the optical response in this section. The nanowire-cone structure [31] has been reported to be an excellent dual-scale structure as an antireflective coating on the silicon surface. The 2D simulation of FDTD solutions is employed to investigate the hybrid structure (nanowire-cone, nanoripple-cone) and micron cone alone due to the computing resources. The detailed modeling information and 2D models are shown in Figure 6. The unit cells of structures all consist of micron cone (base width = 4 µm, height = 5 µm). Several nano cones are coated on the micron cone from a lateral perspective of the nanoripple-cone.

**Figure 7:**
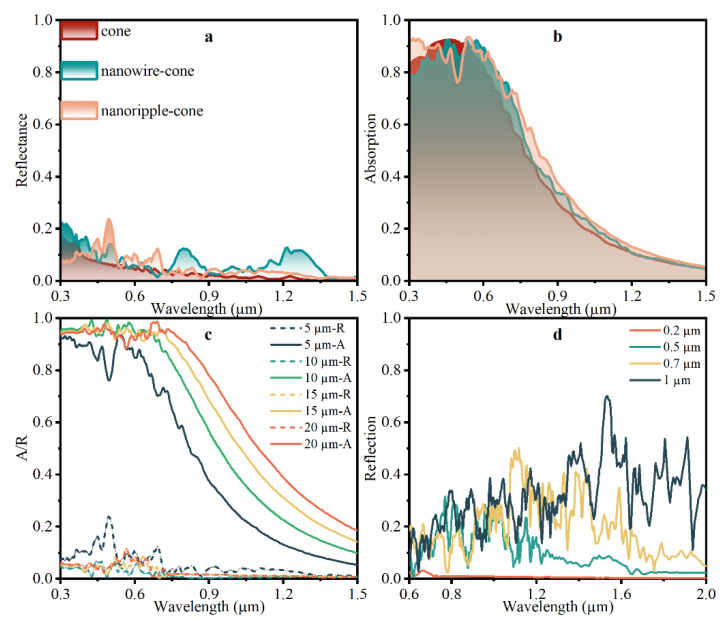
(**a**) Reflectance curves and (**b**) absorption spectra of structures (cone alone, nanowire-cone and nanoripple-cone), as shown in Figure 6. Additionally, optical responses for nanoripple-cone (**c**) with different micro-scale cones and (**d**) with different nano-scale ripples.

The reflectance spectra and absorption spectra for structures shown in Figure 6 are demonstrated in Figure 7a,b. In Figure 7a, the reflectance curve of the nanoripple-cone is analogous to the cone alone, which is almost below 0.1 in the waveband from the visible light region and NIR. Meanwhile, Figure 7b indicates that the absorption property of the nanoripple-cone outperforms the nanowire-cone and cone alone, particularly in the NIR region. Then, the influence of the height of micron cone or nanoripple is deeply explored. The corresponding reflectance curves and absorption spectra for nanoripple-cone structures are shown in Figure 7c. The case is performed for four different heights of micron cones (H = 5, 10, 15, 20 µm) with a fixed nanoscale ripple (height = 200 nm, width = 100 nm). The reflectance decreases as the height of the micron cone increases, though the trend is slight when the height increases to 10 µm. It is significant that the absorption is enhanced with the rising micron cone height. The absorption curve for the 5 µm nanoripple-cone falls at a wavelength of 600 nm, while the 20 µm nanoripple-cone decays at 0.8 µm. The absorption for the 20 µm nanoripple-cone is around 95% in the visible light spectrum. Additionally, the high absorption property is extended to the near-infrared region. In comparison, the absorption for the 20 µm cone alone without nano texture is 70% at the wavelength of 0.8 µm, while the reflectance is about 30%, thus, the benefit of the dual-scale structure is more evident as the wavelength increases. Figure 7d shows the reflectance spectra for four different heights of nanoscale ripple (h = 200 nm, 500 nm, 700 nm, 1 µm) with a fixed micron cone (base width = 7 µm, height = 15 µm). It is evident that the 200 nm ripple-micron cone exhibits the lowest reflectivity, which is close to zero in the wavelength region of 0.6–2 µm. Therefore, a low optical reflectivity of hybrid structure over a wideband spectrum is supposed to be obtained with the increasing micro-scale cone and the decreasing nanoscale of ripple.

**Figure 8:**
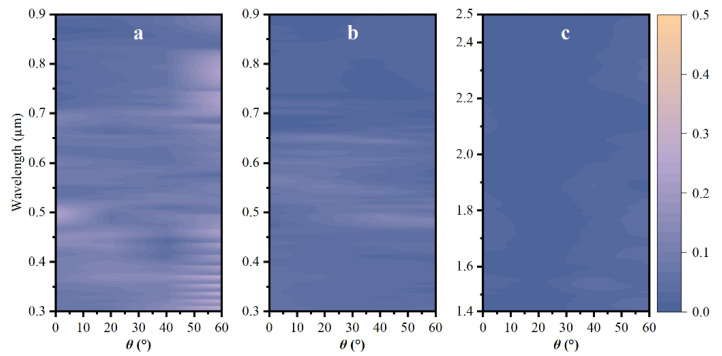
Reflectance spectra for (**a**) the 5 µm nanoripple-cone and (**b**) the 20 µm nanoripple-cone over the incident angle from 0° to 60° as a function of wavelength from 0.3–0.9 µm, as well (**c**) for the 20 µm nanoripple-cone in the NIR region.

The dependence of reflectivity on incident angle θ for the nanoripple-cone is also investigated and the results are shown in Figure 8. The reflectivity for 5 µm nanoripple-cone increases with an increasing incident angle. In contrast, the effect of the incident angle is not remarkable on the 20 µm nanoripple-cone, and the average reflectance is below 3% over the varying angle whether it is in the visible light region or NIR waveband. Besides, the hybrid structure with 20 µm nanoripple-cone outperforms (exhibits lower reflectivity over varying incident angles) the 20 µm cone alone shown in Figure 5d, particularly in the NIR region. The hybrid structure with a higher micron-scale cone is more angle-insensitive than the cone alone due to the emergence of nanoripple. This makes it promising to be applied to more devices based on all-angle black silicon, which shows enhanced efficiency and wide application potential.

We have studied the dependence of reflectivity on the shape and height size of structure fabricated on silicon. The nanoripple-micron cone is proposed to reduce reflectivity and enhance efficiency of devices. The hierarchical texture consists of both nanoscale and micron-scale. The nanoscale ripple, which is smaller than wavelength, has an influence to reduce reflection and enhance light coupling because of the refractive index grating effect [44]. At the air–solid interface, the nano texture works as a medium and the reflective index changes from 0 (for air) to 3.8 (for bulk silicon) as the light is trapped in structures. Moreover, the micron-scale cone works to improve light absorption due to multireflection between cones as the incident light travels [34]. Both two physical mechanisms are beneficial to increase the optical path length of incident light through a device, while providing more photogenerated carriers that can be collected. The above simulation results support that the nanoripple-micron cone is an excellent solution to reduce optical reflectivity and remains as the incident angle varies. The excellent optical response is also extended with an adequate size of micron cone and nanoripple.

### 3.2. Dual-Scale Structure Fabricated by Femtosecond Laser

**Figure 9:**
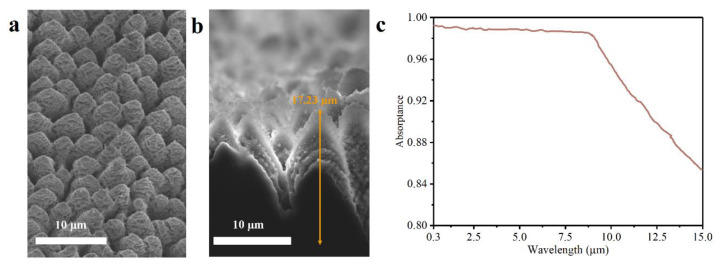
(**a**) The morphology and (**b**) the cross-sectional image of the prepared nanoripple-cone. (**c**) The average corresponding absorption spectrum for three samples treated by the same femtosecond laser parameters, showing approaching results.

Previous works focused on the texture-on-texture way to fabricate dual-scale structures coated on silicon, which is constrained by costs and the processing time. Here, we prepared the nanoripple-cone structure directly by femtosecond laser in ambient air. The morphology and cross-sectional image of black silicon shown in Figure 9a,b result from laser-inducing assisted with laser plasma shockwave cleaning in air, and the measured absorption curve is illustrated in Figure 9c. Nanoscale ripples are coated on the micron cone. The height of the micron cone is in the order of 16.5–18 µm, which is obtained from 20–30 data points on the sample surface. The measured absorption is above 98% in the wavelength range from 0.3–9 µm and the value falls gradually to 85.4% as the wavelength increases to 15 µm. The excellent optical response of the nanoripple-cone extends to the mid-infrared region.

Four mechanisms are working for the light absorption of planar material [34]: (a) intrinsic absorption due to band gap; (b) impurities or lattice defects; (c) lattice vibration; (d) free carriers. As for the textured surface, the fabricated structure is the additional mechanism to reduce reflectivity and enhance light absorption. It is evident that the simulation results are different from the measured curve due to simplified models and neglect of impurity. The optical response for a wavelength below 1.1 µm is mainly governed by geometry structure and band gap of material. The mechanisms of geometry shape and (b)–(d) concluded above are responsible for the optical absorption of wavelength above 1.1 µm. Due to the emergence of air, the oxygen element is doped into silicon and forms a Si-O band, which contributes to an impurity absorption band at about 9 µm due to Si-O stretching vibrations [45]. The previous work also shows that the lattice vibration works for the optical response of a wavelength range from 8 µm to 25 µm [45]. The measured curve is in agreement with the reported mechanisms.

The nanoripple-cone structure, which is fabricated directly by laser-inducing assisted with laser plasma shockwave cleaning in ambient air, is supported to be a preferred cost-effective candidate to get a wideband optical response by simulation and experiments and could be applied to devices for wavebands from visible to mid-infrared, including solar cells, photodetection, SERS, sensing and so on. To get better performance and efficiency, appropriate methods and processing parameters should be elected experimentally. The aforementioned work [43] analyzed that the fabricated nanoripple-cone structures by our method are getting more uniform, denser and higher with increasing cleaning times and reduced laser-cleaning velocity, as well as the better optical absorption. The heights of micron cone and nanoripple depend on laser processing parameters. Besides, the proposed method is also beneficial for the development of multi laser-processing and the evaluation of corresponding experiments.

## 4. Conclusions

A number of simulations and experiments were undertaken to investigate the dual-scale structure for Si-based absorbers in this study. The effect of geometric shape and size on the optical response of structures are studied first. The simulation results indicate that the micron cone structure outperforms (shows lower reflectivity) other micron textures. Additionally, the low reflectance region moves along the spectrum from visible light to NIR with the increasing height size of structure arrays. The dual-scale nanoripple-cone structure is proposed creatively. The low reflectivity is obtained with the increasing size of the micron cone and the decreasing size of nanoripple. Besides, the nanoripple-cone could remain the low-reflectivity property under varying incident angles as the waveband from visible light to NIR. Moreover, the nanoripple-cone structure is fabricated directly by laser-inducing assisted with laser plasma shockwave cleaning in air, which is an economical way instead of a costly texture-on-texture way. The dual-scale structure textured by femtosecond laser shows excellent absorption properties, maintaining absorption above 98% in the waveband of 0.3–9 µm. This study provides useful guidance for the fabrication of B-Si-based optoelectronic devices. Meanwhile, the proposed dual-scale structure and processing method have application potential in enhancing absorption at mid-infrared and THz frequencies.

## Data Availability

The data presented in this study are available on request from the corresponding authors.
